# Immunopathological Response, Histological Changes, Parasitic Burden, and Egg Output in Sheep Naturally Infected by *Dicrocoelium dendriticum*

**DOI:** 10.3390/ani11020546

**Published:** 2021-02-19

**Authors:** Giuseppe Piegari, Paola Pepe, Davide De Biase, Ilaria d’Aquino, Antonio Bosco, Giuseppe Cringoli, Serenella Papparella, Laura Rinaldi, Orlando Paciello

**Affiliations:** 1 Department of Veterinary Medicine and Animal Production, Unit of Pathology, University of Naples “Federico II”, 80137 Naples, Italy; davide.debiase@unina.it (D.D.B.); Ilaria.daquino@unina.it (I.d.); papparel@unina.it (S.P.); paciello@unina.it (O.P.); 2 Department of Veterinary Medicine and Animal Production, Unit of Parasitology and Parasitic Diseases, University of Naples “Federico II”, 80137 Naples, Italy; paolapep04@yahoo.it (P.P.); boscoant@tiscali.it (A.B.); cringoli@unina.it (G.C.); lrinaldi@unina.it (L.R.)

**Keywords:** *Dicrocoelium dendriticum*, FLOTAC, leucocytic inflammation, fibrosis, sheep

## Abstract

**Simple Summary:**

*Dicrocoelium dendriticum*, commonly known as the lancet liver fluke, is a liver parasite that affects ruminants and occasionally other species, including humans. The aim of this study was to investigate the histological modification and the phenotype of inflammatory cells in the livers of sheep naturally infected with *D.dendriticum* and the correlation of these variables with the parasitic burden, egg output, and gross appearance of the liver. We studied twenty-four sheep selected on the basis of positive *D. dendriticum* fecal egg counts. Gross and histological modifications of the liver and the number of adult *D. dendriticum* worms were examined. Macroscopically, the livers were swollen with thickened bile ducts, cholangitis, and fibrosis. Histologically, we observed leucocyte infiltration, bile duct hyperplasia, and fibrosis. Lesions were present in different degrees of severity and were scored. A significant positive association was observed between the number of adult worms recovered from the liver, egg per gram, macroscopic lesions, fibrosis, and bile duct hyperplasia. A significant negative association was observed among these variables and the degree of leukocyte infiltration. The immunohistochemical examination showed a CD3+ predominant cell population in all assessed animals. These findings allow us to better understand the complex mechanism of the host–parasite interaction, in relation to the egg output and parasitic burden in dicrocoeliosis.

**Abstract:**

The aim of this study was to investigate the correlation between infection by *Dicrocoelium dendriticum* (class Trematoda) and the animal host response in terms of macroscopic lesions, the immunopathological response, and histological changes in the livers of naturally infected sheep. Twenty-four sheep were selected on the basis of positive *D. dendriticum* fecal egg counts (FECs). Gross and histological injuries were scored. A positive significant association was observed between the number of adult worms recovered from the liver, FEC, macroscopic lesions, fibrosis, and bile duct hyperplasia. A significant negative association was observed among these variables and the degree of leukocyte infiltration. In addition, immunophenotyping of the inflammatory cells was carried out using primary antibodies against T cell epitopes (CD3+, CD4+, and CD8+), B cell epitopes (CD79α), and the ionized calcium-binding adapter molecule 1 (IBA-1) antigen. Independently of the severity of the *D. dendriticum* infection, the predominant cell population was CD3-positive and associated with lesser numbers of CD79α- and Iba-I-positive cells. An increase in Iba-1-positive cells was observed in the livers of animals with a high worm burden. Our results provide a reference basis to better understand the local immune response in sheep naturally infected by *D. dendriticum* in relation to the FEC and parasitic burden.

## 1. Introduction

The lancet liver fluke *Dicrocoelium dendriticum* is a zoonotic parasite of the bile ducts and gallbladder of different mammalian animal species (mainly ruminants), including humans [1,2,3]. The life cycle of this parasite is complex and involves numerous species of land molluscs and ants as first and second intermediate hosts, respectively [4,5]. In the definitive hosts, *D. dendriticum* spreads directly within liver biliary ducts without affecting other liver structures, such as the liver capsule or liver parenchyma, as in fasciolosis [6]. Dicrocoeliosis is a worldwide zoonotic disease. 

In Italy, dicrocoeliosis is one of the most widely spread hepatic helminth infections in small ruminants [7,8] with high prevalence values (up to 67%) as demonstrated in a study conducted on sheep farms in southern Italy [9]. This parasitic disease is also a cause of high economic losses in the livestock industry due to decreasing the rate of production, impaired female fertility, and slow animal development, as well as liver condemnation (mainly in cattle, sheep, and goats). Furthermore, Dicrocoeliosis on farms is a cause of increasing costs for anthelmintic treatments [10].

Specific clinical symptoms of infestation are not typically manifested, even in severe infections, and, therefore, major lesions, due to liver impairment, are detectable only at post-mortem examination [1,2] and are directly proportional to the parasitic burden [11] and chronic inflammation of the bile duct [12]. The in vivo diagnosis of dicrocoeliosis is routinely performed by the microscopic detection of parasite eggs at coprological examination. In contrast, gross and histological changes and the detection of the adult flukes into the bile ducts are the parameters assessed at post-mortem examination [6]. 

Macroscopically, the main liver injuries are bile duct inflammation, enlarged liver, and fibrosis [11]. In experimentally infected lambs, histopathological changes were characterized by a broad range of injuries, such as periductal fibrosis, ductal reaction, and leukocyte infiltration [13]. Different studies evaluated the phenotypic expression of inflammatory cells in animals experimentally infected by the liver fluke, Fasciola hepatica, [14,15], whereas fewer reports investigated the immunopathological features in animals experimentally infected by *D. dendriticum* [13]. 

To the best of the authors’ knowledge, no study had yet evaluated the local immune response in sheep naturally infected by *D. dendriticum*, and no study had investigated the relationship between the local immune response and other variables, such as hepatic lesions, the parasitic burden, and the egg output, in either naturally or experimentally infected animals. A better understanding of the local immune response in natural infected animals, and its correlation with different variables, such as liver histological changes or the adult parasitic burden, could be useful to address the research toward new vaccine strategies or in further studies on the immunopathology of the parasitic infection. 

In light of these observations, the aim of this study was to unravel the histological modification and the phenotypic expression of inflammatory cells in the livers of sheep naturally infected by *D. dendriticum* and the correlation of these variables with the parasitic burden, egg output, and gross appearance of the liver.

## 2. Material and Methods

### 2.1. Study Farm and Animals 

The study was conducted in the Campania region (southern Italy) on a commercial dairy sheep farm with a known history of parasitic infections by *D. dendriticum*. The animals were on permanent pastures and were treated with moxidectin less than six weeks before the start of the study.

Fecal examinations were performed to select sheep based on the *D. dendriticum* infection status. Specifically, individual fecal samples were collected directly from the rectum, and the fecal egg counts (FECs) were performed with the FLOTAC technique (analytic sensitivity = 1 egg per gram (EPG) of feces), using the zinc sulphate as the flotation solution (specific gravity; s.g. = 1.35) [16]. Twenty-four sheep (age range 2–3 years; non-pregnant females) were selected on the basis of the *D. dendriticum* FEC and divided into four groups of six sheep each as follows: Group A (<30 EPG), Group B (30–100 EPG), Group C (>100 EPG), and Group D (negative).

### 2.2. Gross Examination

The 24 animals were slaughtered, within 48 h post FEC, and the liver, gall bladder, and the first 3 m of small intestine were collected. The study did not require consent or ethical approval according to European Directive 2010/63/EU. The animals were slaughtered in strict accordance with European slaughter regulations (CE n.1099/2009 of 24 September 2009) for the protection of animals at the time of killing (Official Journal of the European Union L 303/1). Permission to obtain the samples was granted from the owner of the abattoir and from the veterinary inspector responsible for the sanitary surveillance.

Liver gross lesions were evaluated using a four-point liver lesions score (LLS) according to Jithendran and Bhat [11]. The main assessed parameters were as follows: the degree of fibrosis, bile duct inflammation, and the worm burden. The gross lesions were evaluated by two independent pathologists (G.P. and O.P.) with a concordance rate of 97%.

### 2.3. Parasitic Burden

During the gross examination, the livers were cut in slices and squeezed by hand in a bowl of tap water to evaluate the parasitic burden. The bowl contents were washed with a stream of water on a sieve (0.18 mm diameter) and transferred into a solution of 70% ethyl alcohol to fix the small liver flukes. The gall bladder and the small intestine were opened, and the flukes present were collected and added to those from the liver washing. The recovered *D. dendriticum* worms were counted under a stereomicroscope.

### 2.4. Histopathological Examination

After the slaughter of the animals, and immediately after the macroscopic examination, representative tissue samples from each animal were collected from the liver. These tissues were fixed in 10% neutral-buffered formalin and dehydrated through graded alcohols before being embedded in paraffin wax. Sections were cut at 5-micron thickness and were stained with hematoxylin–eosin (HE) and Masson’s trichrome [17,18].

A histological scoring system was used considering the most representative lesions included: bile duct hyperplasia, fibrosis and degree of leucocyte infiltration. Severity of fibrosis was graded, based on the ratio between fibrosis and total area examined, into the following categories: 0 (absent), 1 (mild; <10%), 2 (moderate; 10–30%), and 3 (severe; >30%). The ratio was obtained observing five fields at 40× magnification per animal. The level of bile duct hyperplasia was scored into the following categories: 0 (absent), 1 (mild; number of biliary branches sectioned in damaged area < 10), 2 (moderate; 10–15 sectioned branches), and 3 (severe; number of biliary branches sectioned in damaged area > 15).

The degree was obtained observing 10 fields at 400× magnification per animal randomly selected in damaged hepatic areas. The inflammation was graded into the following categories: 0 (absent), 1 (mild inflammation, scattered or forming lymphoid aggregates without expansion of liver parenchyma), 2 (moderate inflammation, leucocytic infiltration with moderate expansion of liver parenchyma), and 3 (severe inflammation, leucocytic infiltration with marked expansion of the liver parenchyma). The degree was obtained observing 10 fields at 200× magnification per animal.

The degree of leucocytic infiltration and bile duct hyperplasia was evaluated by two independent pathologists (G.P. and O.P.) with a concordance rate of 98%. The fibrosis degree was assessed by analyzing the fields with image Pro Plus 7.0 software (Media Cybernetics, Silver Spring, MD, USA) [19].

### 2.5. Immunohistochemical Examination

Paraffin-embedded samples were sectioned (5 μm thick), dewaxed with xylene, hydrated, and irradiated in a microwave oven (maximum power, 800 W) in trisethylenediamine tetraacetic acid buffer (EDTA; 10 mM Tris base, 1 mM EDTA solution, 0.05% Tween 20) pH 9.0, for 10 min. The peroxidase activity was inhibited by immersing the slides in hydrogen peroxide 0.3% in absolute methanol for 20 min. The sections were incubated overnight at 4 °C with primary antibodies:

CD3 (polyclonal rabbit anti-human, T lymphocytes, DAKO A/S, IS503, Glostrup, Denmark) diluted 1:100;

CD79*α* (mouse monoclonal mouse anti-human CD79*α*, B-linage cells clone HM57, DAKO A/S, Glostrup, Denmark) diluted 1:50;

Ionized calcium-binding adapter molecule 1 (IBA-1) (Rabbit polyclonal antibody, cod. n. 019-19741, WAKO, Richmond, United States) diluted 1:600;

CD4 (mouse monoclonal, clone 17D1, VMRD, Pullman, United States), diluted 1:100;

CD8 (PT36B, mouse monoclonal, clone PT36B, VMRD, Pullman, United States), diluted 1:100.

The slides were washed with PBS, then incubated with biotinylated secondary antibody, and labelled with streptavidin biotin for 30 min at room temperature, followed by incubation with streptavidin conjugated to horseradish peroxidase (LSAB Kit, DakoCytomation, Glostrup, Denmark). The reaction was revealed by diaminobenzidine treatment (DakoCytomation, Denmark), and finally the sections were counterstained with Mayer’s hematoxylin. Approximately 20 fields at 20x magnification were evaluated for each section by two independent pathologists (G.P. and O.P.) with a concordance rate of 97%.

According to Costagliola et al. [20], the inflammatory cell immunoreactions for CD3+, CD79*α*, CD4+, CD8+, and IBA-I were scored as follows:

0 (not detected).
1 (percentage of immunoreactive inflammatory cells per section 1–25%).
2 (percentage of immunoreactive inflammatory cells per section 26–50%).
3 (percentage of immunoreactive inflammatory cells per section >50%).

### 2.6. Statistical Analysis

Statistics were computed using SPSS Version 22.0 (IBM Corporation, 2014, Armonk, NY, United States). The positive or negative associations between the EPG, the number of adult worms recovered per sheep on post-mortem examination, and the macroscopic and microscopic liver lesions were evaluated by Spearman’s Rho correlation; *p* < 0.05 was considered significant. To evaluate the differences between groups, an unpaired rank sum test was calculated (two-sided Mann–Whitney U test, with Cronbach’s alpha = 0.05) [21].

## 3. Results

### 3.1. Coprological Analysis and Parasitic Burden

The results of coprological analysis showed *D. dendriticum* FEC values ranging from 6 to 855 EPG. In the twenty-four animals examined in this study, the number of recovered adult worms of *D. dendriticum* ranged from 0 to 7850. In particular, the parasitic burden from the Group A animals ranged from 58 to 109 (mean 88.6 ± 26.3 SD), while those of Groups B and C ranged from 111 to 398 (mean 244.3 ± 94.3 SD) and 890 to 7850 (mean 2639.8 ± 2464.4 SD), respectively. The negative control animals did not show any pathology or parasites, including *D. dendriticum*, in the liver. None of the 24 animals had concomitant infection with *F. hepatica* or other liver or enteric parasites.

Positive significant association was found between the number of adult worms recovered from the liver and EPG values (Rho = 0.963; *p* < 0.01) (Table 1).

### 3.2. Gross Examination

The livers of the control group showed no macroscopic modification. The livers of Groups A and B showed slight to moderate fibrosis with a variable number of white patches on the surface and within the parenchyma. In some livers, the bile ducts were thickened with catarrhal exudate within the lumen, a moderate worm burden, and mild to moderate fibrosis; in others, the cut surface was moderately congested with a low worm burden (Group A: parasitic burden range 58–109; mean 88,6 [±26.3 SD]; Group B: parasitic burden range 111–398; mean 244,3 [±94.3 SD]).

In the livers of Group C, a large number of *D. dendriticum* were detectable inside the bile ducts and gall bladder (parasitic burden range 890–7850; mean 2639,8 [±2464.44 SD]), the organ was swollen, with thickened ducts, cholangitis, whitish spots on the surface, and moderate to severe fibrosis. The Mann–Whitney U test showed a statistically lower degree of gross lesions in Group A compared with those detected in Group B (*p* < 0.05; z score: −2.815) and C (*p* < 0.05; z score: −3.035) and a lower degree in Group B than those detected in Group C (*p* < 0.05; z score: −2.345). Finally, Spearman’s Rho test showed a positive significant correlation between the EPG, parasite burden, and degree of macroscopic lesions (Rho = 0.900; *p* < 0.01). The results are summarized in Table 1.

### 3.3. Histological Examination

The main histopathological lesions in the liver of animals with dicrocoeliosis were represented by various degree of fibrosis, bile duct hyperplasia, and lymphoplasmacytic inflammation. The livers of the control group showed no histological changes. In the livers in Group A (EPG < 30), we observed mild periductal fibrosis, mild bile duct hyperplasia, and severe leukocyte infiltration, mainly located around the septal and inter-lobular bile ducts or forming lymphoid aggregates.

**Figure 1:**
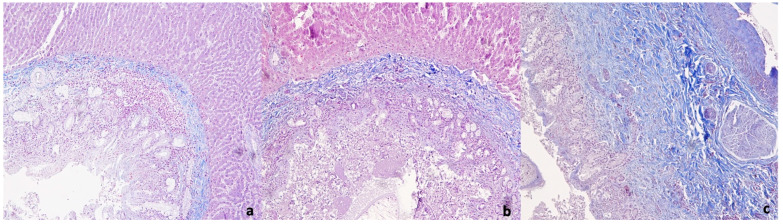
**Representative sections from the liver tissues of sheep** (**a**): Liver section from sheep of Group A (<30 egg per gram (EPG)) showing mild fibrosis and severe leukocyte infiltration; (**b**): liver section from sheep of Group B showing moderate fibrosis and leukocyte infiltration around the bile ducts; (**c**): liver section from sheep of the Group C showing severe fibrosis around the bile ducts and mild leukocyte infiltration. Masson’s trichrome (original magnification 100×).

The livers in Group B (EPG 30–100) showed mild to moderate fibrosis and bile duct hyperplasia. A moderate to severe leukocyte infiltration around the bile ducts, diffusely or forming lymphoid aggregates, and the presence of leukocytes in the liver sinusoids were also seen. Finally, in Group C (EPG > 100), we observed severe fibrosis with the expansion of most bile ducts and portal areas. In this group, the degree of leukocyte infiltration was mild and mainly located around the bile ducts and portal areas. In contrast, the degree of bile duct hyperplasia was severe (Figure 1).

Spearman’s Rho test showed a positive significant correlation between EPG, the parasite burden, and bile duct hyperplasia (Rho = 0.867; *p* < 0.01) and fibrosis (Rho = 0.904; *p* < 0.01). On the other hand, a significant negative correlation (Rho = −0.829; *p* < 0.05) was observed between EPG, parasite burden, fibrosis, biliary duct hyperplasia and the degree of leukocyte infiltration. In addition, the Mann–Whitney U test showed a statistically higher degree of bile duct hyperplasia and fibrosis in Group C than those detected in Group A and control animals; a higher degree of bile duct hyperplasia and fibrosis in Group C than those detected in Group B was also observed. In contrast, the same statistical test showed a lower degree of leukocyte infiltration in Group C compared with those detected in Group A and a lower degree of leukocyte infiltration in Group C compared with those detected in Group B (*p* < 0.05). The results of the histological examination are summarized in Table 1.

### 3.4. Immunohistochemical Evaluation

The inflammatory cell phenotypes were identified based on the staining pattern of antibodies against cell surface proteins. The control animals showed no immunopositivity for T cell (CD3+, CD4+, CD8+) or B cell (CD79α) epitopes. Iba-1-positive cells were observed within hepatic sinusoids in all samples in Group D (the control group). In all infected livers, independently of the severity of the *D. dendriticum* infection or degree of leukocyte infiltration, the predominant cell population was CD3-positive associated with lesser numbers of CD79α- and Iba-I-positive cells. Statistical analysis showed a higher percentage of CD3-positive cells compared with CD79α and Iba-I positive population in all three assessed groups (*p* < 0.05).

In animals of Group A (EPG < 30), a CD3+, CD79α, and Iba-I positive population was found around the bile ducts, in small multifocal aggregates or forming lymphoid follicles. In animals of the Groups B (EPG 30–100) and C (EPG > 100), CD3+ and CD79α-positive cells were observed in the hepatic sinusoids, around the inter-lobular bile ducts, septal bile ducts, and forming lymphoid follicles. Lymphoid aggregates were composed of CD79α-positive cells surrounded by CD3-positive lymphoid cells. CD3-positive cells were often observed within the lymphoid follicles. Subpopulations of CD4+ and CD8+ T cells were found in all three assessed groups; CD4-positive cells were observed scattered diffusely around the bile ducts or associated with lymphoid aggregates.

**Figure 2:**
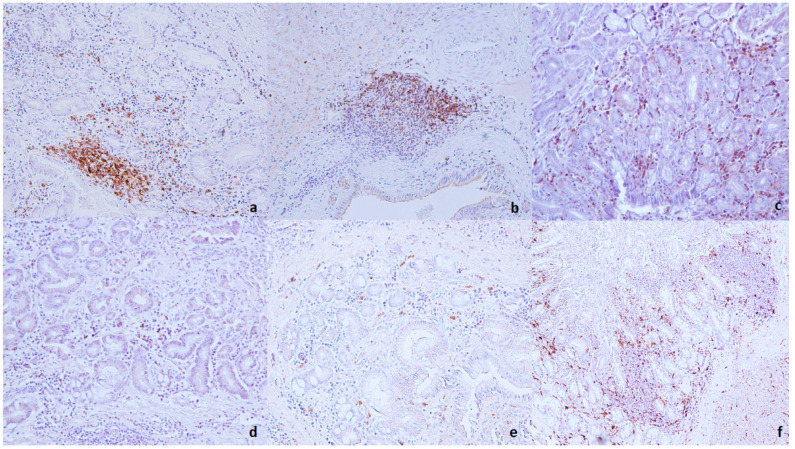
**Liver** (**a**): Lymphoid aggregate composed of CD79-positive cells associated with a moderate number of CD-79-positive lymphocytes around the hyperplastic bile duct (liver from Group A); (**b**): CD3-positive cells in the periphery of the lymphoid aggregate (liver from Group B); (**c**): CD3+ cells scattered diffusely around the hyperplastic bile ducts (liver from Group C); (**d**): a moderate number of CD8+ cells around the bile ducts (liver from Group A); (**e**): a moderate number of CD4+ cells around hyperplastic bile ducts (liver from Group B) (immunohistochemistry, HRP method, original magnification 200×); (**f**): a moderate number of ionized calcium-binding adapter molecule 1 (IBA-1)-positive cells scattered around the bile ducts and within follicular aggregates (liver from Group A) (immunohistochemistry, HRP method, original magnification 100×).

CD8+-positive cells were often observed around the hyperplastic bile ducts or in small focal aggregates. Finally, in all animals in Groups B (EPG 30–100) and C (EPG > 100), Iba-1-positive cells were located scattered diffusely around the hyperplastic bile ducts or within lymphoid follicles and hepatic parenchyma (Figure 2). Spearman’s Rho test showed no correlation between these variables and the degree of leukocyte infiltration. In addition, the Mann–Whitney U test showed no statistical differences for CD3+- and CD79α-positive cells among Groups A (EPG < 30), B (EPG 30-100), and C (EPG > 100). However, the same test showed a statistically higher percentage of Iba-I-positive cells in group C (EPG > 100) compared with those detected in group A (EPG < 30) (*p* < 0.05; z score: −3.317). The results of the immunohistochemistry are summarized in Table 2 and Table 3.

## 4. Discussion

Few studies have been conducted thus far on the immune-pathological aspects of sheep naturally infected by *D. dendriticum* compared to under experimental conditions. Experimentally infected sheep, bred in pasture systems and without additional feed, are much more susceptible to parasitic infection; in contrast, sheep bred in the traditional system and fed with high protein diets can implement a better immune response to reduce the parasite impact [22]. Therefore, information obtained from sheep experimentally infested needs to be validated in naturally infected sheep.

### 4.1. Coprological Analysis and Parasitic Burden

Overall, the statistical analysis showed a positive correlation between the *D. dendriticum* burden and the EPG in all infected animals. As a consequence, a high number of recovered *D. dendriticum* adult worms was related to a high number of parasite eggs eliminated into the environment. A significant positive correlation between worm burden and liver gross pathology was found. With regard to the first observed correlation, similar results were obtained by Campo et al. [23] in lambs experimentally infected with different doses of *D. dendriticum*. This was in line with other studies on the correlation between egg elimination and the parasitic burden in animals naturally and experimentally infected by the lancet liver flukes [22,24,25].

Similar results were also obtained for other parasitic infections. Radfar et al. [26] showed a strong correlation between the EPG of *Fasciola hepatica* and the burden of worms in the liver of cattle that were naturally infected, whereas Rinaldi et al. [27] showed a significant positive correlation between the FEC and gastrointestinal strongyle worm burden in goats. Finally, with regard to the correlation between the *D. dendriticum* worm burden and the macroscopic lesions in the liver, similar results were obtained by Jithendran and Bhat [11] in sheep naturally infected by *D. dendriticum*.

### 4.2. Gross and Histological Examination

To the best of the authors’ knowledge, this is the first study to investigate the relationship among the egg excretion, worm burden and macroscopic lesions and the histological lesion and local immune response of the liver of sheep naturally infected by *D. dendriticum*. Overall, the pathological features of the liver and local immune response observed in the four groups of sheep included in the study (Group A < 30 EPG; Group B = 30–100 EPG; Group C > 100 EPG; Group D = negative) were different in terms of bile duct hyperplasia, fibrosis, degree of leucocyte infiltration, and inflammatory cell phenotypes. In particular, statistical analysis showed that the EPG and parasite worm burden positively correlated with biliary duct hyperplasia and fibrosis, as well as negatively correlated with the degree of leukocyte infiltration.

As *D. dendriticum* is a parasite that completes its life cycle within the bile ducts, the positive association between bile duct hyperplasia and the parasitic burden could be explained by mechanical parasite irritation due to the suckers of the adult flukes [28,29]. In this context, previous studies conducted on liver rodents showed a self-proliferation of cholangiocytes following bile duct ligation, suggesting a specific correlation between bile duct damage and hyperplasia [30,31]. Severe bile duct damage can also determine a trans-differentiation of hepatocytes into cholangiocytes and consequent ductal hyperplasia [31,32,33]. 

Regarding the correlation between the EPG, parasite burden, bile duct hyperplasia, and fibrosis, similar results were obtained by Marcos et al. [34] in a study conducted on cattle naturally infected by *F. hepatica.* This positive correlation is not surprising considering the strong relationship between the ductal reaction and fibrosis in livers. Indeed, although the secretion of pro-fibrotic and anti-inflammatory mediators by macrophages is considered an important fibrogenesis component, a concomitant inhibition of the cholangiocyte proliferation determines a reduction in fibroblastic proliferation and fibrosis in the liver [31,35].

Finally, the parasitic burden, bile duct hyperplasia, and fibrosis were negative correlated with the degree of leucocyte infiltration. These findings could suggest a key role of fibrotic mechanisms related to the parasite, reducing the interaction between the parasite antigens and the immune system and modulating the inflammatory response. Furthermore, as previously reported in other parasites, it is possible to suppose a role of the parasite itself in the modulation of the host immune responses [36]. However, further study will be needed to confirm this hypothesis.

### 4.3. Immunohistochemical Evaluation

Immunohistochemical examination showed CD3- and CD79α-positive cells in the liver of all three positive assessed groups; the CD3 and CD79α percentages of positive cells were not correlated with fibrosis, bile duct hyperplasia, the degree of leucocyte infiltration or the worm burden, suggesting that both phenotypical different cells participated in the local immune host response independently of the infestation or leucocyte infiltration degree. Statistical analysis showed a higher percentage of CD3-positive cells compared with the CD79α- and Iba-I-positive population in all three assessed groups.

A sub-population of CD4-positive cells and a lesser percentage of CD8-positive cells were also observed in all assessed groups. These findings, taken together, suggest a prevalent cellular-mediated response in the livers infected by *D. dendriticum,* even in animals with a lower leucocyte infiltration degree. Studies have identified these T lymphocyte sub-populations in lambs experimentally infected by *D. dendriticum* [13]; in addiction, previous studies reported a CD4+ T sub-population in the early response to *F. hepatica* in rodent livers [37].

Interestingly, the detection of CD79α-positive cells indicated a concomitant humoral immune response, likely in reaction to somatic and excretory antigens of the parasites. The role of somatic antigens of *D. dendriticum* involved in antibody responses has been investigated in infected cattle and rats [6]. Local humoral immunity induces a broad range of responses, such as superoxide generation, the release of inflammatory mediators and interference with parasite growth, diffusion, and adherence [6]. However, the protective role of antibodies in dicrocoeliosis must be further elucidated.

Lymphoid cells were often observed in lymphoid aggregates or lymphoid follicles, composed of CD79α-positive cells surrounded by lymphoid cells labelled positively for CD3 polyclonal antibodies. Similar structures were observed in lambs experimentally infested with different doses of metacercariae, mainly at 180 days post infection [13]. Finally, the Mann–Whitney U test showed no statistical differences for CD3- and CD79α-positive cells among Groups A, B, and C. However, the same test showed a statistically higher percentage of Iba-1-positive cells in group C compared with those detected in group A.

Iba-1 is currently considered a pan-macrophage biomarker, and, consequently, its concomitant increase may reflect an increase in the macrophage population in livers with higher worm burden and egg expression [38]. This increase could reflect the central role of macrophages in the modulation of the inflammation response and architectural remodeling of tissue, which are distinctive features highlighted in the present study in the livers of sheep with a higher worm burden and egg excretion [39].

Overall, the local immune response that was observed in the present study appears qualitatively similar to that previously reported by Ferreras-Estrada et al. [13] in lambs experimentally infested by *D. dendriticum*, suggesting similar immune responses in both experimentally infested lambs and naturally infested sheep.

## 5. Conclusions

Our results provide a reference basis to better understand the liver immune response in naturally infected sheep, in relation to the egg output and liver worm burden. The observed correlation between the FEC and macroscopical and histological lesions of the liver could suggest the use of the FEC technique as an indirect index of liver damage due to natural infection by *D. dendriticum*. However, further studies will be needed to confirm this hypothesis.

Finally, our results, if confirmed on a larger scale, could be used to improve the therapeutic protocols for the management of sheep affected by *D. dendriticum.* Indeed, as we discover more regarding the mechanisms of host–parasite interactions and the complex response for protection against parasitic infections, we are also laying the foundation to address the research toward new vaccine strategies and immunomodulatory therapies.

## Figures and Tables

**Table 1:** Results of the coprological analysis (EPG values), post-mortem examination (worm burden), and scoring assigned to macroscopic and histological alterations. * EPG: egg per gram; ** LLS: liver lesions score; *** BDH: bile duct hyperplasia; **** LI: leucocyte infiltration.

Group	EPG *	Worm Burden	LLS **	Fibrosis	BDH ***	LI ****
	12	69	1	1	1	3
	12	76	1	1	1	3
A	6	84	1	1	1	2
	9	58	1	1	1	3
	18	136	1	1	1	3
	24	109	2	1	1	3
	36	164	2	2	1	3
	30	248	2	1	2	2
	36	398	2	2	2	2
B	30	111	3	1	2	2
	36	228	3	1	1	2
	96	317	3	2	2	2
	150	1310	3	3	3	1
	120	890	3	3	3	1
C	156	1327	4	3	3	1
	228	3328	3	3	3	1
	144	1134	4	3	3	1
	855	7850	4	3	3	1
	0	0	0	0	0	0
	0	0	0	0	0	0
D	0	0	0	0	0	0
	0	0	0	0	0	0
	0	0	0	0	0	0
	0	0	0	0	0	0

**Table 2:** Results of the immunohistochemical examination.

Group	CD3+	CD4+	CD8+	Cd79 α	IBA-1
	3	2	1	2	1
	3	2	1	1	1
A	3	2	1	3	1
	2	1	1	1	1
	3	2	1	2	1
	3	1	2	1	1
	3	1	2	2	1
	2	2	1	1	1
B	2	1	1	2	2
	3	2	1	1	1
	3	2	2	2	2
	3	2	1	2	1
	3	1	2	2	1
	3	2	1	2	2
C	3	2	1	2	2
	3	2	1	2	2
	2	1	1	2	2
	3	2	1	2	2

Scoring system applied for the inflammatory cell’s immunoreactions: 0 (not detected); 1 (percentage of immunoreactive inflammatory cells per section 1–25%); 2 (percentage of immunoreactive inflammatory cells per section 26–50%); 3 (percentage of immunoreactive inflammatory cells per section >50%).

**Table 3:** Median for each group for each inflammatory cell’s phenotype.

Group	CD3+	CD4+	CD8+	Cd79 α	IBA-1
A (EPG < 30)	3	2	1	2	1
B (EPG 30–100)	3	2	1	2	1
C (EPG > 100)	3	2	1	2	2

Scoring system applied for the inflammatory cell’s immunoreactions: 0 (not detected); 1 (percentage of immunoreactive inflammatory cells per section 1–25%); 2 (percentage of immunoreactive inflammatory cells per section 26–50%); 3 (percentage of immunoreactive inflammatory cells per section >50%).

## Data Availability

All relevant data is listed in the manuscript.
